# The Coexistence of Antiphospholipid Syndrome and Systemic Lupus Erythematosus in Colombians

**DOI:** 10.1371/journal.pone.0110242

**Published:** 2014-10-24

**Authors:** Juan-Sebastian Franco, Nicolás Molano-González, Monica Rodríguez-Jiménez, Yeny Acosta-Ampudia, Rubén D. Mantilla, Jenny Amaya-Amaya, Adriana Rojas-Villarraga, Juan-Manuel Anaya

**Affiliations:** 1 Center for Autoimmune Diseases Research (CREA), School of Medicine and Health Sciences, Universidad del Rosario, Bogotá, Colombia; 2 Mederi, Hospital Universitario Mayor, Bogotá, Colombia; Beth Israel Deaconess Medical Center, United States of America

## Abstract

**Objectives:**

To examine the prevalence and associated factors related to the coexistence of antiphospholipid syndrome (APS) and systemic lupus erythematosus (SLE) in a cohort of Colombian patients with SLE, and to discuss the coexistence of APS with other autoimmune diseases (ADs).

**Method:**

A total of 376 patients with SLE were assessed for the presence of the following: 1) confirmed APS; 2) positivity for antiphospholipid (aPL) antibodies without a prior thromboembolic nor obstetric event; and 3) SLE patients without APS nor positivity for aPL antibodies. Comparisons between groups 1 and 3 were evaluated by bivariate and multivariate analysis.

**Results:**

Although the prevalence of aPL antibodies was 54%, APS was present in just 9.3% of SLE patients. In our series, besides cardiovascular disease (AOR 3.38, 95% CI 1.11–10.96, *p* = 0.035), pulmonary involvement (AOR 5.06, 95% CI 1.56–16.74, *p* = 0.007) and positivity for rheumatoid factor (AOR 4.68, 95%IC 1.63–14.98, *p* = 0.006) were factors significantly associated with APS-SLE. APS also may coexist with rheumatoid arthritis, Sjögren's syndrome, autoimmune thyroid diseases, systemic sclerosis, systemic vasculitis, dermatopolymyositis, primary biliary cirrhosis and autoimmune hepatitis.

**Conclusions:**

APS is a systemic AD that may coexist with other ADs, the most common being SLE. Awareness of this polyautoimmunity should be addressed promptly to establish strategies for controlling modifiable risk factors in those patients.

## Introduction

Autoimmune diseases (ADs) are chronic conditions initiated by the loss of immunological tolerance to self-antigens, and they represent a heterogeneous group of disorders that affect specific target organs or multiple systems. Because of the chronic condition of these diseases, they constitute a significant burden on the health care system, with direct and indirect economic costs and quality of life impairment [1]–[4].

The fact that ADs share several subphenotypes, physiopathological mechanisms, environmental factors, and genetic factors has been called the “autoimmune tautology” and indicates that ADs share common physiopathological mechanisms and a common origin [1]–[9].

The clinical evidence of the autoimmune tautology highlights the coexistence of distinct ADs within an individual, which corresponds to polyautoimmunity, defined as the presence of more than one AD in a single patient [1], [10]. When three or more ADs coexist, this condition is called multiple autoimmune syndrome (MAS) [11]. Factors significantly associated with polyautoimmunity include female gender and familial autoimmunity (i.e., the presence of diverse ADs in multiple members of a nuclear family) [10], [12]. Polyautoimmunity represents the effect of a single genotype on diverse phenotypes [1].

Several subphenotypes are shared by ADs, including cutaneous involvement (i.e., photosensibility, alopecia, Raynaud's phenomenon), arthralgia and arthritis, fatigue, and even cardiovascular disease (CVD) [1], [2]. Polyautoimmunity has been reported in most ADs, including systemic lupus erythematosus (SLE) [10], [13], in which one of the most frequent coexistent disease is antiphospholipid syndrome (APS) [13]. Therefore, the purposes of this study were to examine the prevalence and associated factors of the coexistence of SLE and APS in a cohort of Colombian patients with SLE, as well as to discuss on the coexistence of APS with other ADs.

## Patients and Methods

### Study population

A cross-sectional analytical study was conducted in 376 Colombian patients with SLE. The subjects have been systematically followed at the Center for Autoimmune Diseases Research (CREA) in Bogota, Colombia. All of the subjects fulfilled the 1997 update of the American College of Rheumatology (ACR) classification criteria for SLE [14].

The patient socio-demographic and cumulative clinical and laboratory data, as well as a household description, were obtained by interview, standardized report form, physical examination and chart review as previously reported [15]. The data were collected in an electronic and secure database. The socio-demographic variables included age at SLE onset, disease duration, educational and socioeconomic status, current occupation, smoking habits, coffee consumption, expositional factors and physical activity. The age at onset (AOD) of the disease was defined as the first subjective experience of the symptom(s) and/or sign(s) described in any of the items of the classification criteria [16]. The duration of disease was considered as the difference between the AOD and the date of the first participation in the study. The educational level was recorded as the number of years of education and was divided into two groups (equal to or more than 9 years and less than 9 years of education) based on the “General Law of Education” in Colombia [17], [18]. The socioeconomic status was categorized on the basis of national legislation and was divided into low (1 and 2), intermediate (3) and high (4–6) status [19]. The smoking habits were assessed as never smoking; 1–6, 6–15, and >15 packages/year; or cessation of cigarette consumption. Coffee intake was asked as yes or not and measured in cups per day (i.e., 1–2, 2–4, >4).

### Ethics statement

This study was performed in compliance with Act 008430/1993 of the Ministry of Health of the Republic of Colombia, which classified it as minimal-risk research. All of the patients voluntarily accepted to participate in the study by reading and signing the informed consent. The institutional review board of the Universidad del Rosario approved the study design.

### Clinical variables

Clinical and laboratory variables were registered as present or absent at any time during the course of the disease as previously reported [15], [20]. In particular, lupus nephritis was defined as active urinary sediment, or proteinuria >500 mg/24 h or positive renal biopsy (ISN/RNP). Pulmonary compromise was considered in the presence of pulmonary hypertension, or pulmonary embolism or diffuse alveolar hemorrhage. Pleuritis was defined as the presence of pleural rub and/or effusion and/or typical pleuritic pain, and confirmed by chest X-ray or computed tomography. Neurologic involvement included the presence of seizures without any other definable cause, or psychosis lacking any other definable cause, or other conditions such as peripheral neuropathy, stroke, transverse myelitis, chorea, or other central nervous system lesions directly attributable to SLE in the absence of other causes. Autoimmune hemolytic anemia (hematocrit <35%, reticulocyte count >4%, and positive Coombs test), leukopenia (white cells <4000/mm3) and thrombocytopenia (platelets <100000/mm3) were also assessed. Other manifestations such as polyautoimmunity [1], [10], MAS [11], familial autoimmunity, and familial autoimmune disease [12] were also included.

CVD represents a broad spectrum of manifestations (i.e., subphenotypes) such as hypertension (HTN), myocardial infarction (MI), peripheral vascular disease (PVD), and cerebrovascular accidents (i.e., stroke). Since atherosclerosis (AT), the usual cause of CVD, starts when the endothelium becomes damaged, and is considered to be an autoimmune-inflammatory disease, CVD was assessed by characterizing the following five subphenotypes: HTN, defined as having blood pressure ≥140/90 mm Hg recorded as an average of two measurements, with at least a 15-minute interval between the measurements or the use of any antihypertensive medication [21]; a history of stroke; a coronary event [i.e., unstable angina, MI]; a thrombotic event (other than MI and carotid involvement, requiring anticoagulant treatment); and carotid disease (by the Doppler criteria or intima-media thickness ≥0.9). The traditional risk factors assessed and included were smoking [22], type 2 diabetes mellitus [23] and dyslipidemia [24].

Concerning pharmacological treatment, the current or past use of steroid therapy (i.e., prednisolone, methylprednisolone, deflazacort), antimalarials (i.e., cloroquine, hydroxychloroquine), immunosupressors (i.e., azathioprine, mycophenolate mofetil and cyclophosphamide) and biological therapy (i.e., rituximab) were also recorded.

### APS polyautoimmunity assessment

All of the patients were systematically assessed and classified for the presence of the following: 1) confirmed APS according to the 2006 update of the classification criteria [i.e., a thromboembolic event and positivity for anti-cardiolipin (aCL) IgG/IgM in medium or high titer, anti-beta 2-glycoprotein 1 (B2GPI) IgG/IgM antibodies or lupus anticoagulant (LAC), on two or more occasions] [25]; 2) positivity for antiphospholipid (aPL) antibodies (i.e., aCL IgG/IgM, B2GPI IgG/IgM or LAC) without a prior thromboembolic nor obstetric event; and 3) SLE patients without APS nor positivity for aPL antibodies.

### Laboratory measurements

The relevant laboratory variables associated with SLE were registered. Patients underwent measurement of the following antibodies by ELISA using commercial kits (QUANTA Lite, INOVA Diagnostics, San Diego, CA, USA): anti-double strand DNA antibodies (anti-dsDNA), precipitating antibodies to extractable nuclear antigens (ENAs: Sm, U1-RNP, Ro/SS-A, La/SS-B), aCL IgG and IgM, B2GPI IgG and IgM antibodies and rheumatoid factor (RF) IgM. The positive cut-off values were >300 UI for anti-dsDNA; >40 UI aCL (IgG and IgM), and B2GPI (IgG and IgM); >20 UI ENAs; and >6 UI for RF. Additionally, the antinuclear antibodies (ANAs) were measured by indirect immunofluorescence on HEp-2 cell (NOVA Lite™ Hep-2 ANA kit, INOVA Diagnostics, San Diego, CA, USA), according to the manufacturer's instructions. Other autoantibodies, including lupus anticoagulant, anti-Scl 70, -centromere, -mitochondrial, and -smooth muscle antibodies, were recorded. White blood cell and platelet count, hemoglobin levels, mean corpuscular volume, complement (i.e., C3 and C4 levels), venereal disease research laboratory results, and creatinine levels were extracted from each patient's clinical record.

### Statistical analysis

First, univariate descriptive statistics were performed. The categorical variables were described by the frequencies. The quantitative continuous variables were described as the mean and standard deviation, as well as the median and interquartile range. The comparisons between group 1 (i.e., confirmed APS) and 3 (i.e., SLE patients with neither APS nor positive aPL autoantibodies) in relation to the other categorical variables, were assessed by chi square tests or Fisher's exact tests when facing small sample sizes. The comparisons between the two groups in relation to the continuous variables were performed with the Kruskal-Wallis test. A logistic regression model with APS and SLE polyautoimmunity as the dependent variable was fit (group 1 vs. group 3). As the independent factors, the model included the variables that were statistically significant in the bivariate analyses and those variables that were biologically plausible. The model was adjusted by gender and duration of the disease. The adequacies of the logistic models were assessed using the Hosmer–Lemeshow goodness-of-fit test. The Nagelkerke R2 (i.e., the pseudo-R2) was used to estimate the percentage of variance explained by the models. The adjusted odds ratios (AOR) were calculated with 95% confidence intervals (CI). The Wald statistical test was used to evaluate the significance of the individual logistic regression coefficients for each independent variable. The statistical analyses were performed in R 3.0.2 [26].

## Results

Of the 376 patients with SLE, 91.8% were women. Thirty-five patients fulfilled the classification criteria for APS, with a prevalence of 9.3%, representing the second most common polyautoimmunity, preceded by autoimmune thyroid disease, which disclosed a prevalence of 12%. The range of time elapsed from the SLE onset to the diagnosis of APS was 1 to 7 years. All of the patients had SLE prior to the diagnosis of APS. Out of the 35 patients with APS-SLE, 51.4% had a prior thromboembolic event, 40% pregnancy loss and 8.5% had both. Among the non-criteria manifestations, 9 (26%) patients had pulmonary involvement, of whom 6 had pulmonary hypertension and 3 pulmonary embolism, 6 (17%) patients had thrombocytopenia, and 5 (14%) livedo reticularis. Regarding CVD, this was present in 22 (63%) patients, of whom 18 had thrombosis and 10 hypertension (29%). There were 2 additional patients with prior thromboembolic and/or obstetric manifestations and positivity for aPL below medium or high titers that were excluded from further analysis, but in whom a “seronegative” APS could not be rule out. The most frequently found aPL antibody was LAC, with a prevalence rate of 51.5%, followed by aCL IgM with 40% and IgG with 33%. B2GPI IgG and IgM were positive in 11% and 13% of the patients, respectively. Of the SLE patients, 60% had neither APS nor positivity for aPL antibodies. The characteristics of the cohort are illustrated in Table 1. Socioeconomic status, smoking and coffee intake had no significant influence of the coexistence of SLE-APS. Although SLE disease activity index (SLEDAI) was calculated upon study entry, this variable was not included in the analysis.

**Table 1:** Demographic and clinical characteristics of 376 patients with SLE.

*Characteristic*	*Median, range*
Age(y)	36, 25–46
Age at SLE onset (y)	27, 19–38
Duration of disease (y)	5, 2–11
***Sociodemographic characteristics***	***%(n/N)***
Female	91.8 (345/376)
LEL	13.8 (51/369)
Low SES	24.2 (86/355)
Ever smoking	36.3 (134/369)
Coffee intake	61.8 (225/364)
***Clinical variables***	***%(n/N)***
Female	91.8 (345/376)
Arthritis	74.7 (281/376)
Malar rash	44.4 (167/376)
Photosensitivity	57.2 (215/376)
Oral ucers	30.6 (115/376)
Alopecia	49.7 (187/376)
Serositis	30 (113/376)
Renal involvement	44.9 (168/376)
Neurological involvement	40.9 (154/376)
Pulmonary involvement	10.4 (39/376)
Haematological involvement	79.1 (295/376)
CVD	38.5(145/376)
Pregnancy loss	20.5 (70/342)
Poliautoimmunity	31.1 (112/376)
AITD	11.97 (45/376)
SS	9.04 (34/376)
RA	6.12 (23/376)
MAS	7.98 (30/376)
Familial autoimmunity	19.68 (74/376)
***APS assessment***	***%(n/N)***
Group 1 (APS)	9.3 (35/376)
Group 2 (aPL antibodies)	30.8 (116/376)
Group 3 (SLE without APS nor aPL)	59.8 (225/376)
***Medication***	***%(n/N)***
Steroid therapy	87.3 (281/322)
Antimalarials	86 (277/322)
Immunosupressors^a^	51.1 (164/321)
***Laboratory findings***	***%(n/N)***
ANA (+)	100 (376/376)
Anti dsDNA (+)	66.1 (236/357)
Anti Sm (+)	39.9 (144/361)
Anti Ro (+)	49.45 (180/364)
Anti La (+)	25.6 (93/363)
Anti RNP (+)	44.5 (161/362)
LAC (+)	51.45 (71/158)
aCL IgG (+)	33.3 (120/360)
aCL IgM (+)	39.8 (140/352)
B2GPI IgG (+)	11.3 (33/293)
B2GPI IgM (+)	13.5 (39/288)
RF IgM (+)	42.1 (120/285)
Hypocomplementemia^b^	66.4 (209/315)

APS: antiphospholipid syndrome; aCL: anticardiolipin antibodies; AITD: autoimmune thyroid disease; ANA: antinuclear antibodies; B2GPI: anti-β2 glycoprotein I antibodies; CVD: cardiovascular disease; LAC: lupus anticoagulant; LEL: low educational level; MAS: multiple autoimmune syndrome; RA: rheumatoid arthritis; RF: rheumatoid factor; SS: Sjögren's síndrome; SES: socioeconomic status; y: years.

a Immunosupressors based on treatment with mycophenolate mophetil and/or cyclophosphamide and/or azathioprine.

b Hypocomplementemia defined as the presence of low C3 or C4.

### Factors associated with APS polyautoimmunity

There was no difference in the patient age, AOD or duration of SLE. CVD, Sjögren's syndrome (SS), pulmonary involvement and positivity for RF were risk factors significantly associated with APS. The presence of alopecia was negatively associated with APS (Table 2).

**Table 2:** Factors associated with APS in SLE (bivariate analysis).

	*SLE + APS*	*SLE*		
*Variable*	*%(n/N)*	*%(n/N)*	*OR (95% CI)*	*p*
Gender	91.4 (32/35)	92.9 (211/227)	0.81 (0.22–2.9)	0.746
CVD	62.2 (23/37)	36.1 (82/227)	2.74 (1.42–6.1)	0.002
Abortion	46.8 (15/32)	13.9 (29/308)	4.97 (2.45–11.83)	<0.001
SS	25.7 (9/35)	7.5 (17/227)	3.88 (1.77–10.47)	<0.001
Alopecia	28.6 (10/35)	55.5 (126/227)	0.30 (0.15–0.70)	0.003
Pulmonary involvement	25.7 (9/35)	9.7 (22/227)	2.97 (1.38–7.73)	0.006
RF IgM (+)	57.1 (16/28)	34.5 (59/171)	2.29 (1.12–5.55)	0.02

APS: antiphospholipid syndrome; OR: odds ratio; 95% CI: 95% confidence interval; CVD: cardiovascular disease; RF: rheumatoid factor; SLE: systemic lupus erythematosus; SS: Sjögren's syndrome.

### The adjusted effects of the risk factors for APS polyautoimmunity

As expected, CVD was associated with APS-SLE. The main subphenotypes associated with CVD in APS-SLE were thrombosis and hypertension. Additional variables influencing this condition were pulmonary involvement and positivity for RF, regardless of gender and duration of the disease. Alopecia was confirmed to be negatively associated with APS (Table 3). A second logistic regression model was fit to evaluate the effect of aPL alone (i.e., group 2) on the expression of SLE without APS nor positivity for aPL antibodies (i.e., group 3). An association between aPL and RF was observed, but not with clinical features.

**Table 3:** Adjusted factors associated with APS in SLE polyautoimmunity (multivariate analysis).

*Characteristics*	*β*	*AOR*	*95% CI*	*p* *
CVD	1.218	3.38	1.11–10.96	0.035
Alopecia	−1.556	0.21	0.06–0.64	0.009
Pulmonary involvement	1.62	5.06	1.56–16.74	0.007
RF IgM (+)	1.543	4.68	1.63–14.98	0.006

AOR: adjusted odds ratio; 95% CI: 95% confidence interval; CVD: cardiovascular disease; RF: rheumatoid factor.

*Variables statistically significant are shown.

## Discussion

In our series, the coexistence of APS with SLE was 9.3% and was associated with a distinctive subphenotype characterized by a higher risk of CVD, pulmonary involvement and positivity for RF.

The interplay between SLE and APS has been analyzed over the years, since APS was initially not considered to be a systemic AD and was thought to occur only in patients with SLE [27]–[29]. Furthermore, APS has been found to be associated with systemic manifestations (e.g., non-thrombotic renal disease, migraine, livedo reticularis, pulmonary hypertension, MI) and laboratory features (e.g., positive ANAs, hypocomplementemia) also present in patients with SLE, complicating the clear differentiation between these entities [27]–[29]. However, rather than being the spectrum of the same disease, the coexistence of APS and SLE is a reflection of complex disease traits and highlights the common mechanisms shared by ADs [1], [4].

The association between CVD and SLE mortality was first established by Urowitz et al. [30], with the description of the bimodal pattern of mortality in SLE patients, with an early death due to active disease and infections, and a late death secondary to CVD. Despite a reduction in SLE mortality over the years, CVD remains prevalent among these patients, affecting up to 28% of patients after four decades of disease duration [31], [32]. Traditional risk factors have been described for CVD in SLE (e.g., HTN, dyslipidemia) [33]. However, Esdaile et al. [34] demonstrated that these factors do not fully account for the accelerated AT in these patients, suggesting that there are factors related with the disease involved in the development of CVD. Among the novel risk factors described, polyautoimmunity with APS has been associated with CVD. In fact, APS has been reported as a non-traditional risk factor for CVD in SLE [15]. Other studies have reported similar results, as well as an increased accrual of damage measured by the SLE Damage Index [35]–[38]. Different mechanisms involving the role of aPL in the process of AT have been described, and these include overexpression of tissue factor, annexin I and II, cytokines and other mediators of endothelial dysfunction [39]–[41]; oxidative perturbations and mitochondrial dysfunction [42]; and cross-reactivity with oxidized low-density lipoproteins, accelerating their influx into macrophage and AT development [43]. Recently, Perez-Sanchez et al. [44] found a close relationship between patients with APS and those with polyautoimmunity of SLE and APS in the groups of genes belonging to the cluster of AT, highlighting the role of novel risk factors in the development of CVD among these patients.

The pulmonary involvement described in SLE and APS may be similar, and include pulmonary hypertension, pulmonary embolism and diffuse alveolar hemorrhage [45]–[47]. In our results, the association with pulmonary involvement was driven by the presence of pulmonary hypertension. aPL has been associated with the development of pulmonary hypertension in patients with SLE [48]–[51]. The positivity for RF seem to have a dual role in autoimmunity with a protective effect by interfering with the binding complement system to immune complexes, or a harmful effect by enhancing immune mediated tissue damage [52]. In SLE, positivity for RF has been found to have a protective role in the development of lupus nephritis [53]–[55]. In our results, we found that the positivity for FR IgM was associated with the coexistence of APS and SLE, as well as with the positivity for aPL antibodies. Similarly, Spadaro et al. [52] previously reported the relationship between different isotypes of RF in patients with APS and SLE polyautoimmunity. Positivity for RF IgG was negatively associated with serosal and hematological involvement, and negatively correlated with aCL IgG titer, suggesting that RF IgG is related with the absence of APS; on the other hand, RF IgM was associated with aCL IgM positivity, implying a possible relation with APS [52]. The interplay of RF isotypes in ADs and polyautoimmunity requires further investigation.

As already mentioned, APS was primarily recognized in patients with SLE and was, therefore, considered to be an overlap syndrome (i.e., “secondary” APS). The major difference between polyautoimmunity and overlap syndrome is that the former is the presence of two or more well-defined autoimmune conditions (i.e., overt clinical manifestations mediated by T or B cell responses) fulfilling the validated classification criteria whereas the latter is the partial presence of signs and symptoms of diverse ADs [1]. An overlap syndrome may evolve towards a well-defined phenotype or remain as an undifferentiated AD for years (i.e., the presence of some subphenotypes) [1]. The international consensus statement on an update of the classification criteria for definite APS advised against using the term “secondary” APS because no difference was found in the clinical consequences of aPL antibodies among the patients in this category (Evidence Level I) [25]. The consensus is that, rather than distinguishing patients by “primary” and “secondary” APS, documenting the coexistence of SLE (or other ADs) is more advantageous for classification [25]. In agreement with this argument, we have proposed the term "polyautoimmunity" instead of secondary AD to refer to the coexistence of two or more ADs [10].

Besides SLE, APS has been described in most ADs, including rheumatoid arthritis, SS, autoimmune thyroid diseases, systemic sclerosis (SSc), systemic vasculitis, dermatopolymyositis, primary biliary cirrhosis (PBC) and autoimmune hepatitis (AIH) [35]–[37], [56]–[89]. Moreover, the coexistence of APS with other ADs may be associated with a modified clinical course, especially in SSc (i.e., associated with pulmonary arterial hypertension and MI), PBC (i.e., higher Mayo risk score) and AIH (i.e., associated with cirrhosis and disease activity) (Table 4). Therefore, APS should be addressed in patients with the aforementioned ADs.

**Table 4:** Polyautoimmunity in APS.

*AD*	*APS*	*aCL % (n/N)*		*LAC*	*B2GPI*	*Commentary*	*Ref.*
	*% (n/N)*	*IgG*	*IgM*	*% (n/N)*	*% (n/N)*		
**SLE**	6.5 (44/679)	-	-	-	-	The patients with SLE/APS polyautoimmunity were more likely to die from cardiovascular disease (i.e., myocardial infarction, acute coronary syndrome). These patients had a higher SDI score and poorer survival, which did not reach statistical significance.	[35]
	19.2 (5/26)	25 (5/20)		27.8 (5/18)	-	The SLE patients with intracranial hemorrhage had polyautoimmunity, including APS, more frequently than did the controls. Thrombocytopenia was found to be an independent risk factor for intracranial hemorrhage.	[56]
	19 (19/100)	53 (53/100)		2 (2/100)	84 (84/100)	IgG aCL and B2GPI correlated with a previous history of arthritis.	[57]
	8.4 (8/95)	13.4 (13/95)	2.1 (2/85)	-	-	The occurrence of aCL positivity was significantly associated with venous/arterial thrombosis and abortion. Thrombocytopenia was more frequent among the aCL positive patients; however, it did not reach statistical significance.	[58]
	12.4 (16/129)	15.5 (20/129)	13.9 (18/129)	-	10.1 (13/129)	In a mestizo cohort of SLE patients, aCL positivity was significantly associated with venous/arterial thrombosis and fetal loss.	[59]
	9.2 (12/130)	18.5 (24/130)		-	-	The occurrence of aCL positivity was present before the diagnosis of SLE and prior to the development of the clinical features of APS. The aCL positivity was associated with a higher number of the ACR criteria and early AOD of SLE as well as cutaneous, serosal, neuropsychiatric and renal involvement.	[60]
	12 (20/166)*	-	-	-	-	Polyautoimmunity with APS increased the risk of pregnancy loss, especially after week 20 of gestation.	[61]
	28.4 (50/176)	-	-	-	-	APS and damage (measured by SDI) were significantly associated with higher mortality in SLE patients.	[36]
	14.1 (28/198)	36.9 (73/198)	17.7 (35/198)	21.7 (43/198)	-	The independent predictor factors for diminished survival with SLE duration of 15 years were AOD, renal involvement and APS polyautoimmunity.	[37]
**RA**	3.6 (3/84)	8.3 (7/84)	2.4 (2/84)	-	7.1 (6/84)	The prevalence of aPL antibodies in RA was reported. No correlation was found with the clinical features.	[62]
	-	12.4 (12/97)	11.3 (11/97)	1 (1/97)	12.4 (12/97)	There was a negative association between aPL antibodies and anti-CCP antibodies.	[63]
	-	20.2 (35/173)	15.6 (27/173)	-	-	The presence of aCL antibodies was associated with rheumatoid nodules. The patients with aCL antibodies had more cutaneous manifestations (e.g., livedo reticularis, purpura, cutaneous ulcers); however, this association was not significant.	[64]
	-	8.9 (15/168)	4.2 (7/168)	5.9 (10/168)	-	The RA patients with positivity for aPL antibodies and high levels of homocysteine might be at a higher risk for thrombotic events.	[65]
	-	6.8 (7/102)	0.9 (1/102)	-	-	A significant correlation was found between the aCL and anti-CCP antibody levels.	[66]
	-	11.9 (22/184)	4.3 (8/184)	3.8 (7/184)	-	The RA patients with positivity for aCL and a history of arterial/venous thrombosis were found to have lower levels of free protein S.	[67]
**SS**	4.1 (3/74)	32.4 (24/74)	6.8 (5/74)	10.8 (8/74)	4.1 (3/74)	The aPL-antibody positive patients were associated with polyautoimmunity. They had peripheral neuropathy and the presence of hypergammaglobulinemia.	[68]
	3 (3/100)	1 (1/100)	3 (3/100)	9 (9/100)	5 (5/100)	LAC was associated with a significantly higher risk for deep venous thrombosis, stroke and/or myocardial infarction. No significant association was found with the clinical features.	[69]
	3.1 (4/130)	10.8 (14/130)	6.2 (8/130)	9.2 (12/130)	-	The presence of aPL antibodies was associated with a higher prevalence of ANAs positivity.	[70]
	1.0 (4/402)	4.7 (19/402)	1.5 (6/402)	4.7 (19/402)	-	APS is an infrequent, but not exceptional, polyautoimmunity in SS.	[71]
	-	12.7 (8/63)	6.3 (4/63)	-	-	The presence of aCL antibodies was associated with RNP positivity. No relationship was found with the clinical features or extra glandular manifestations.	[72]
**AITD**	-	35.5 (11/31)	35.5 (11/31)	-	-	There was an increased incidence of aCL in patients with AITD. None of the patients had clinical features of APS.	[73]
	-	43.8 (57/130)	10 (13/130)	-	-	The prevalence of aPL is increased in AITD. There was no correlation between aPL titer and the titer of thyroid autoantibodies.	[74]
	-	10.1 (7/69)	5.8 (4/69)	-	-	The patients with aCL did not exhibit a higher titer of thyroid autoantibodies.	[75]
	-	10.5 (2/19)	10.5 (2/19)	-	-	The patients with Hashimoto's thyroiditis had an increased incidence of aCL. There was no correlation with thyroid autoantibodies titer.	[76]
**SSc**	2.7 (3/108)	11.1 (12/108)	2.7 (3/108)	-	6.5 (7/108)	The SSc patients with aCL positivity were associated with pulmonary hypertension. The patients with PAH had higher titers of aCL; however, this association was not significant.	[77]
	-	19.1 (13/68)	20.6 (14/68)	-	-	Anticentromere antibodies, anti-ScL-70 and smoking were significantly associated with severe ischemia and amputation in SSc, whereas no relationship was found with aCL.	[78]
	-	12.2 (10/82)	3.7 (3/82)	-	-	No association was found between aCL and the clinical features of SSc.	[79]
	-	20.8 (10/48)	35 (17/48)	23.8 (5/21)	-	The presence of IgM aCL was significantly associated with higher cutaneous involvement (e.g., the number of lesions, plaques and body areas affected) and ANA and RF positivity in the patients with localized SSc compared to the controls.	[80]
	-	17.5 (20/40)	5 (2/40)	5 (2/40)	50 (20/40)	The aPL-antibody patients had a tendency to Raynaud's phenomenon, cutaneous ulcerations and pulmonary hypertension; however, this correlation was not significant.	[81]
	-	8.6 (9/105)	11.4 (12/105)	-	-	The SSc patients with aCL were significantly associated with ischemia or myocardial necrosis (i.e., on the electrocardiogram and/or scintigraphic findings). There was a higher prevalence of digital pitting scars, acroosteolysis and pulmonary involvement; however, it did not reach statistical significance.	[82]
	1.4 (1/72)	9.7 (7/72)		-	-	No correlation was found between aPL antibodies and the clinical features of SSc.	[83]
**Systemic vasculitis**	6.3 (12/144)	8 (12/144)		5.6 (8/144)	-	The presence of aPL antibodies might influence the clinical course for systemic vascularity.	[84]
**DM/PM**	3.1 (3/97)	-	-	-	-	The prevalence of polyautoimmunity with APS was reported in a cohort of DM/PM.	[85]
	-	2.7 (1/36)	5.6 (2/36)	-	-	The prevalence of aCL in ADs was reported	[86]
**PBC**	1.4 (1/31)	-	-	-	-	The prevalence of MAS in the patients with AIH/PBC polyautoimmunity was evaluated. AITD was found to be the most prevalent AD. There was no correlation with the therapy response.	[87]
	-	27.3 (27/99)	27.3 (27/99)	-	2 (2/99)	IgG aCL were significantly associated with the presence of cirrhosis, a higher Mayo risk score and thrombocytopenia.	[88]
**AIH**	-	38.9 (23/59)	23.7 (14/59)	-	3.4 (2/59)	IgG aCL were significantly associated with the presence of cirrhosis and disease activity (in the clinical features and the laboratory).	[89]

AD: autoimmune disease; aCL: anticardiolipin antibodies; AIH: autoimmune hepatitis; AITD: autoimmune thyroid disease; anti-CCP: anti-cyclic citrullinated peptides antibodies; AOD: age at onset of disease; APS: antiphospholipid syndrome; B2GPI: anti-beta 2 glycoprotein I antibodies; DM/PM: dermatopolymyositis; LAC: lupus anticoagulant; MAS: multiple autoimmune syndrome; PBC: primary biliary cirrhosis; SDI: systemic lupus erythematosus damage index; SLE: systemic lupus erythematosus; SS: Sjögren's syndrome; SSc: scleroderma; RA: rheumatoid arthritis; RF: rheumatoid factor.

*Correspond to 166 pregnancies in 125 women followed in the Hopkins Lupus Cohort.

Although our study sample size was not negligible, as a cross-sectional study, it would have been more valuable to have had an appropriate follow up to address the association of APS and SLE polyautoimmunity by the inclusion of other measurements such as antibodies isotypes (e.g., B2GPI IgA [90], RF IgG), serologic biomarkers and genetic markers.

## Conclusions

APS is considered a systemic AD, and may coexist with other ADs, SLE being the most frequent. The coexistence of APS and SLE is associated with a more severe subphenotype characterized by a higher CVD rate and pulmonary involvement. CVD is a major cause of morbidity and mortality in SLE patients. Novel risk factors related to autoimmunity are recognized because the traditional risk factors do not completely explain the high CVD rates. APS polyautoimmunity should be addressed in every SLE patient to identify the higher risk population and encourage prevention strategies and early therapeutic intervention. APS should be included as a non-traditional risk factor for CVD in SLE and other ADs in further studies.
